# Healthcare services relaxing natural selection may contribute to increase of dementia incidence

**DOI:** 10.1038/s41598-022-12678-4

**Published:** 2022-05-25

**Authors:** Wenpeng You, Renata Henneberg, Maciej Henneberg

**Affiliations:** 1 Biological Anthropology and Comparative Anatomy Unit, School of Biomedicine, The University of Adelaide, Adelaide, SA 5005 Australia; 2 Institute of Evolutionary Medicine, University of Zurich, Zurich, Switzerland

**Keywords:** Evolutionary genetics, Evolutionary theory

## Abstract

Ageing and genetic traits can only explain the increasing dementia incidence partially. Advanced healthcare services allow dementia patients to survive natural selection and pass their genes onto the next generation. Country-specific estimates of dementia incidence rates (all ages and 15–49 years old), Biological State Index expressing reduced natural selection (*I*_*s*_), ageing indexed by life expectancy e_(65)_, GDP PPP and urbanization were obtained for analysing the global and regional correlations between reduced natural selection and dementia incidence with SPSS v. 27. Worldwide, *I*_*s*_ significantly, but inversely, correlates with dementia incidence rates for both all ages and 15–49 years old in bivariate correlations. These relationships remain inversely correlated regardless of the competing contributing effects from ageing, GDP and urbanization in partial correlation model. Results of multiple linear regression (enter) have shown that *I*_*s*_ is the significant predictor of dementia incidence among all ages and 15–49 years old. Subsequently, *I*_*s*_ was selected as the variable having the greatest influence on dementia incidence in stepwise multiple linear regression. The *I*_*s*_ correlated with dementia incidence more strongly in developed population groupings. Worldwide, reduced natural selection may be yet another significant contributor to dementia incidence with special regard to developed populations.

## Introduction

Dementia is not one specific disease, but a syndrome that affects patient’s ability to perform everyday activities due to a chronic or progressive deterioration of memory, thinking and behaviour^1^. Worldwide, around 50 million people are being affected by Alzheimer's (60–70%), vascular diseases (20–30%), Lewy bodies and frontotemporal dementia^1,2^. Worldwide, dementia not only affects individual patients whose human rights and freedom are unnecessarily restricted and subject to social stigma, but also has tremendous impact on families, caregivers, and society^1,3^.

Although extensive studies have been conducted to explore the etiological factors, only ageing and genetic susceptibility are constantly postulated as the risk factors^4,5^. Fox^6^ has hypothesized that, from evolutionary medicine perspective, humans have been living in a mismatched environment, which may be a risk factor for the increase of dementia onsets. Nevertheless, increasing evidence has shown that chronic health conditions (e.g. diabetes, obesity, hypertension, hearing impairment and cerebrovascular lesions), poor lifestyles (e.g. tobacco smoking, lack of exercise, gluten and meat eating) and psychosocial factors, such as less social engagement and depression are potential risk factors of dementia^7–10^. Recently, it has been shown that excessive alcohol consumption, traumatic brain injury, and air pollution could be responsible for dementia initiation^11^.

The World Health Organization (WHO) has estimated that, in 2021, worldwide, there were more than 55 million people living with dementia. With nearly 10 million new cases every year and the increasing proportion of older people in the population, the number of people living with dementia is expected to rise to 78 million in 2030 and 139 million in 2050^1,12^. Similar trends were also reported in a number of individual countries, such as United Kingdom, China, Australia and United States. Population ageing has been the only risk factor used to explain this increase of annual incidence at a population level^1^. Almost all the studies have indicated that the increase of a population segment aged 65+ is contributing to the increasing presence of dementia.

M. Henneberg and J. Piontek advanced the Biological State Index (*I*_*bs*_) based on the calculation of a population specific fertility and mortality data^13,14^. The *I*_*bs*_ measures probability for an average person in a population to have an opportunity to pass their genes to the next generation. A number of publications co-authored by M. Henneberg have revealed that this probability in different modern populations varies between 63.5% (Burkina Faso) and 99.4% (Iceland and Cyprus), and the average was 92.8%^15–18^.

It is a common sense that advanced healthcare services have allowed more and more people to survive to the age of reproduction and continue through their reproductive age span successfully. From the perspective of Darwinism, the underlying reason is that advanced healthcare services have relaxed natural selection leading to low mortality and low fertility^19^. With the greater portion of a population participating in the reproduction, a concern has been raised that the chance for deleterious genes/mutations being passed onto their next generations has been and will be increasing^20^. The consequence of this accumulating process is that a human population is subject to increasing incidence of non-communicable diseases. Dementia is an umbrella term for a number of non-communicable diseases, some with genetic traits, which can be passed onto the next generation.

The genetic causes of dementia have two forms: (1) directly causing dementia, including Alzheimer's, vascular, Lewy bodies and frontotemporal diseases; (2) causing comorbidities (chronic diseases: diabetes^17,21^, obesity^15,16^, hypertension, mental health disorders). Modern healthcare services have made it possible that people carrying genes/mutations contributing to dementia diseases or its comorbidities are free of dementia presence for all their life, or suffer from mild dementia. These people can reproduce and pass their dementia related genes/mutations to next generations. The portion of a population able to participate in reproduction determines the level of the deleterious genes to be inherited by the next generation. Inspired by these concepts we obtained population specific empirical and macro-level data to examine, from a worldwide perspective, whether populations with higher level of relaxed natural selection have greater dementia incidence.

## Material and method

### Data sources

The dependent variable are population specific incidence rates of “Alzheimer's disease and other dementias (dementia hereafter)” published in the 2020 by the Institute for Health Metrics and Evaluation^22,23^. The estimate of dementia incidence is expressed as the number of people per 100,000 who were newly diagnosed with dementia in the 2019.

In this cross-sectional study, natural selection is the essential part of the study method, and it is primarily related to reproduction. Therefore, for data analysis the dementia incidence rates (1) for all ages and (2) only for 15–49 years old were extracted.

The predicting variable is a current population specific level of natural selection measured by the Biological State Index, (*I*_*bs*_) and its derivative, the index of opportunity for selection (*I*_*s*_) extracted from previous publications^15,16,18^.

The level of reduced natural selection is measured with the Biological State Index (*I*_*bs*_) which was calculated with the population specific fertility and mortality data published by United Nations (2008) and WHO (2012) respectively^24,25^. The *I*_*bs*_ presents the probability that an average individual born into a population is able to fully participate in the reproduction of the next generation giving them an opportunity to pass their genes/mutations to their offspring^13–15^. Considering that the prime driver of Darwinian fitness (adaptive success) is the variance of reproductive success^20,26^, the opportunity for selection (*I*_*s*_) was calculated with the formula, *I*_s_ = (1 − *I*_bs_)/*I*_bs_. This was considered as the independent variable^15,18,20^.

Except for ageing, the two other variables (GDP and urbanization) which have been associated with dementia were also included into data analyses as potential competing variables. Considering the delayed effects of these predicting factors on the dementia onset in 2019, they were backdated for 4–5 years.

1. Ageing is expressed with life expectancy at 65 years old (Life e_(65)_, 2010–2015). These data are regularly published by the United Nations. This study does not take life expectancy at birth because dementia is more common in people over the age of 65, although it can also affect younger people. Another consideration for us to include Life e_(65)_ is that the biopsychosocial functioning of people starts to decline from that age.
2. GDP PPP is expressed in per capita purchasing power parity in 2015 US dollars published by the World Bank^27^. GDP PPP has been associated with prevalence of dementia^1,12^, and it also influences the level of healthcare services that includes screening for dementia and their treatments.
3. Urbanization is expressed by the percentage of population living in urban areas in 2015 as published by the World Bank^27^. Urbanization entails a high level of education, but a poor lifestyle, for example, lack of exercise and social engagement, consumption of food with few nutritional benefits, more gluten and meat eating, air pollution, and consuming more salt, fat, sugar and alcohol. Urban lifestyle has been postulated as a complex risk factor for chronic diseases^28^.

We extracted a list of 204 populations with dementia incidence in 2019, and then we downloaded population specific I_s_ life expectancy e_(65)_, GDP PPP and urbanization before matching them with the dementia incidence list. A set of the population specific data for 204 countries was obtained and stored in the Microsoft Excel® for data analyses. For some populations, the estimate of one or the other variable was missing, thus specific analyses have sample sizes varying from 182 to 204. Each population was treated as an individual study subject and all of their available information was analysed.

In order to demonstrate the universal predicting effect of reduced natural selection on dementia incidence, the populations were grouped for further correlation analyses based on: (1) the WHO geographic regions^29^; (2) the World Bank income classifications^30^; (3) the United Nations gross national income (GNI) classifications^31^; (4) the strong contrast in terms of geographic distributions, per capita GDP PPP levels and cultural backgrounds to get seven population groupings: Asia Cooperation Dialogue (ACD)^32^, the Asia–Pacific Economic Cooperation (APEC)^33^, the Arab World^33^, Population with English as the official language (extracted from personal knowledge and experience), Latin America and the Caribbean (LAC)^34^, Organization for Economic Co-operation and Development (OECD)^33^, and Southern African Development Community (SADC)^35^. In these analyses, we only included those populations for which we could access their data for the specific groupings. Except for the population with English as the official language, all the other population listings were sourced from their respective official websites before matching them with the list of populations with dementia incidence.

### Data analysis

To examine the relationships between the relaxation of natural selection and dementia incidence in different data analysis models, the analysis proceeded in four (4) steps^36–39^:

1. GeoNames, MS, TomTom (©, powered by Bing) was applied to integrate populations into the geographic map in the Excel (®MicroSoft 2016) depending on their locations. The darkness of colour for their areas in the map varies with their level of *I*_*s*,_ the opportunity for natural selection. For mapping clarity and larger number of populations to be included in the map, the population label on the map is indicated as the ISO code of the population instead of the full name.
  Scatter plots were also prepared for exploring and visualizing the correlation between the opportunity for natural selection and dementia incidence at a population level. Data quality and variable distributions can be examined in scatter plots as well. Mapping selection opportunity, calculations of mean *I*_*s*_, sample size and standard deviation, and producing scatter plots Excel (Microsoft® 2016) have been done using raw data (not log-transformed).
  The 204 countries were also grouped as per the WHO geographic classifications for comparing mean *I*_*s*_ in different regions.
  Before running correlation analyses all data were logarithmed (ln), which reduced possible curvilinearity of regressions and data non-homoscedasticity due to their distributions.
2. Bivariate correlations (Pearson’s and nonparametric, Spearman’s “rho”) were conducted to examine the strength and direction of the correlations between all variables. Bivariate correlations were also performed for each data set of population groups to further explore and compare the correlations between *I*_*s*_ and dementia incidence.
3. Partial correlation of Pearson’s moment-product correlation was performed to examine the correlation between selection opportunity and dementia incidence while the competing variables (ageing, GDP PPP and urbanization) were kept statistically constant.
  We alternated the four variables (*I*_*s*_, ageing, GDP PPP and urbanization) as the predicting variable to explore its relationship with dementia incidence while controlling for all the other three variables. Thus, we could analyse and compare the levels of the independent correlations between dementia incidence and each of four potential risk factors^40,41^.
4. Standard multiple linear regression (enter model) was conducted to analyze the correlations between dementia incidence and each of the four predicting variables. Subsequently, stepwise linear regression was performed to select the predictor(s) having the best influencing effects on dementia incidence.

In the above Steps 2–4, dementia incidence rates were alternated as the dependent variables for data analyses, and results were reported in parallel.

Bivariate correlations, Pearson’s moment-product partial correlation and multiple linear regressions were performed in SPSS v. 27 (Chicago Il USA). The significance was reported when p was below 0.05, but the stronger significance levels, such as *p* < 0.01 and *p* < 0.001 were also indicated in this study. Regression analysis criteria were set at probability of F to enter ≤ 0.05 and probability of F to remove ≥ 0.10.

All the above data analysis methods were performed in accordance with the relevant guidelines and regulations.

### Ethics approval and informed consent

All the data supporting our findings in this paper were freely downloaded from the United Nation agencies’ websites. No ethical approval or written informed consent for participation was required.

### Consent for publication

Not applicable.

## Results

**Figure 1:**
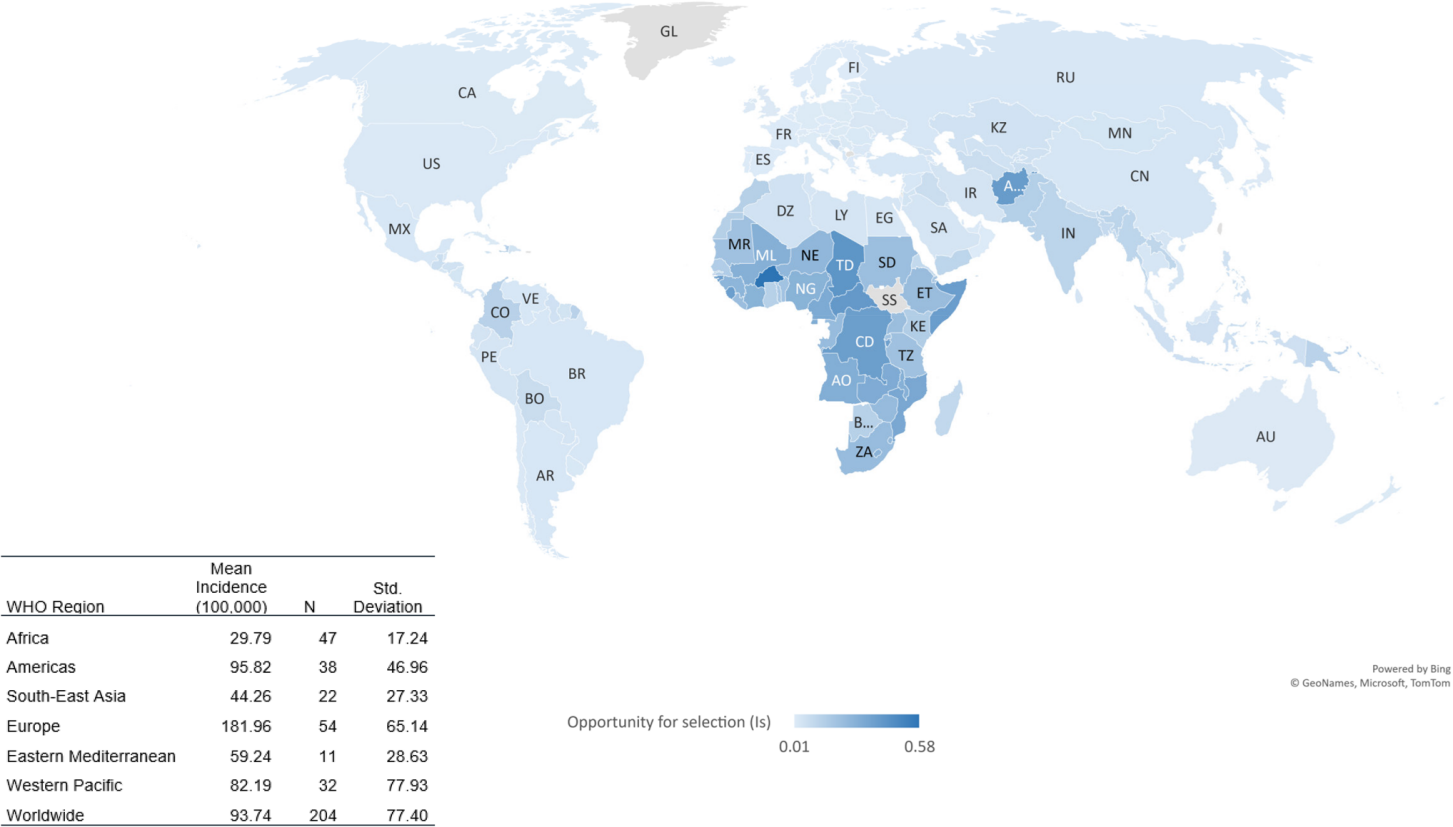
The magnitudes of the opportunity for natural selection *I*_*s*_ in different countries.

Figure 1 shows that, currently every population has very low magnitude of *I*_*s*_ because natural selection in each population has been reduced significantly by the advanced healthcare services. Worldwide, the *I*_*s*_ ranges between 0.01 (Iceland and Cyprus) and 0.58 (Burkina Faso), and the arithmetic mean is 0.09, which has been approximately 2.5 times reduced from 100 to 150 years ago (*I*_*s*_ = 0.22)^20^. In other words, worldwide, humans’ opportunity for natural selection with the advent of modern medicine has decreased approximately 2.5 times in the past 100–150 years. Among the six WHO Regions, the highest and lowest *I*_*s*_ lied in Africa (*I*_*s*_ = 0.23) and Europe (*I*_*s*_ = 0.02).

**Figure 2:**
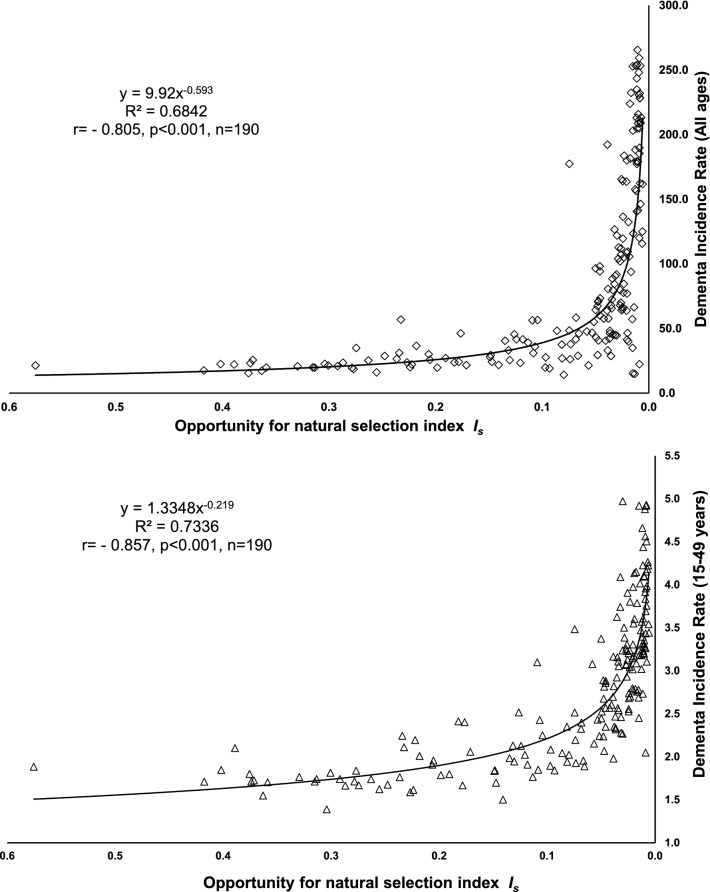
The relationships between opportunity for selection *I*_*s*_ and dementia incidence in populations of full age range and in 15–49 years old population segments.

Figure 2 shows that, inversely, *I*_*s*_ was in power correlation with dementia incidence in populations of all ages (r = − 0.805, *p* < 0.001, n = 190) and in 15–49 years old (r = − 0.857, *p* < 0.001, n = 190).

Table 1 shows that *I*_*s*_ is a significant predicting variable of dementia incidence in bivariate analyses. Worldwide, dementia incidence has significant and strong, but inverse, associations with *I*_*s*_ in both Pearson’s r (r = − 0.827, *p* < 0.001) and Spearman’s rho (r = − 0.857, *p* < 0.001). Additionally, in both data analysis models, dementia incidence is in significant and moderate, but positive, associations with ageing, GDP PPP and Urbanization respectively (r > 0.500, *p* < 0.001). Table 1 also presents correlations between all variables.

**Table 1:** Pearson’s r (above the diagonal) and Spearman’s rho (below the diagonal) between the opportunity for selection (*I*_*s*_) and dementia incidence.

	All ages						15–49 years old				
	Dementia incidence	*I*_*s*_	Ageing	GDP PPP	Urban		Dementia incidence	*I*_*s*_	Ageing	GDP PPP	Urban
Dementia incidence	1	− 0.827***	0.773***	0.724***	0.518***	Dementia incidence	1	− 0.857***	0.752***	0.793***	0.555***
*I*_*s*_	− 0.830***	1	− 0.827***	− 0.866***	− 0.594***	*I*_*s*_	− 0.861***	1	− 0.827***	− 0.866***	− 0.594***
Ageing	0.776***	− 0.835***	1	0.754***	0.560***	Ageing	0.752***	− 0.827***	1	0.754***	0.560***
GDP PPP	0.732***	− 0.877***	0.767***	1	0.698***	GDP PPP	0.793***	− 0.866***	0.754***	1	0.698***
Urbanization	0.522***	− 0.647***	0.645***	*0.*738***	1	Urbanization	0.555***	− 0.594***	0.560***	*0.*698***	1

Significance level: *p < 0.05; **p < 0.01; ***p < 0.001; Sample size range: 175–204.

Data sources: Dementia Incidence expressed as the number of new onsets of Alzheimer's disease and other dementias published by the Institute for Health Metrics and Evaluation (IHME) in 2020; *I*_*s*_ measuring the magnitude of the opportunity for natural selection, extracted from the previous publication by You et al. https://doi.org/10.1111/eva.12523; Ageing expressed with life expectancy at 65 years old (2010–2015) published by the United Nations; GDP PPP expressed in purchasing power parity in 2015 US dollars published by the World Bank; Urbanization expressed in percent of population living in urban areas in 2015 published by the World Bank.

Table 2 suggests that *I*_*s*_ is a significant risk factor for dementia incidence regardless of the competing effects of ageing, GDP PPP and urbanization. This is evidenced by examining the relationship between dementia incidence and *I*_*s*_, ageing, GDP PPP and urbanization respectively which were alternated as the predicting variable while the other three variables were kept statistically constant. In populations of all ages, dementia incidence significantly correlates with *I*_*s*_ (r = − 0.429, *p* < 0.001) and ageing (r = 0.278, *p* < 0.001). However, dementia incidence correlates with *I*_*s*_ stronger than it does with ageing where it is on the edge of significant level (z = 1.58, *p* = 0.0571). In population segments of 15–49 years old, dementia incidence is in significant correlation with I_s_ (r = − 0.452, *p* < 0.001), ageing (r = 0.126, *p* < 0.001) and GDP PPP (r = 0.160, *p* < 0.05). Dementia incidence, however, is in significantly stronger correlation with I_s_ than with ageing (z = 1.58, *p* < 0.001) and GDP PPP (z = 2.98, *p* < 0.001).

**Table 2:** Comparison of partial correlation coefficients between dementia incidence and each variable when the other three variables were kept statistically constant.

Variables	r	*p*	r	*p*	r	*p*	r	*p*
**Dementia incidence rate in populations of all ages**								
*I*_*s*_	− 0.429	< 0.001	–	–	–	–	–	–
Ageing	–	–	0.278	< 0.001	–	–	–	–
GDP PPP	–	–	–	–	− 0.018	0.800	–	–
Urban	–	–	–	–	–	–	< 0.05	0.763
**Dementia incidence rate in population segments of 15–49 years old**								
*I*_*s*_	− 0.452	< 0.001	–	–	–	–	–	–
Ageing*	NA	NA	NA	NA	NA	NA	NA	NA
GDP PPP	–	–	–	–	− 0.160	< 0.05	–	–
Urban	–	–	–	–	–	–	< 0.01	0.912

Significance level: *p < 0.05; **p < 0.01; ***p < 0.001; sample size range: 170 for all analyses.

Data sources: Dementia Incidence expressed as the number of new onsets of Alzheimer's disease and other dementias published by the Institute for Health Metrics and Evaluation (IHME) in 2020; *I*_*s*_ measuring the magnitude of the opportunity for natural selection, extracted from the previous publication by You et al. https://doi.org/10.1111/eva.12523; Ageing expressed with life expectancy at 65 years old (2010–2015) published by the United Nations; GDP PPP expressed in purchasing power parity in 2015 US dollars published by the World Bank; Urbanization expressed in percent of population living in urban areas in 2015 published by the World Bank.

Table 3 shows that *I*_*s*_ is the only variable which has significant influence on dementia incidence in the linear regression model (enter) for the populations with full age ranges (β = − 0.620, *p* < 0.001) and segments of 15–49 years old (β = − 0.679, *p* < 0.001). In the subsequent stepwise multiple linear regression, *I*_*s*_ has been selected as the variable which has the significant predicting effect on dementia incidence in populations with full age ranges (R^2^ = 0.685) and in population segments of 15–49 years old (R^2^ = 0.738). For the segments of 15–49 years old, GDP PPP significantly correlates with dementia incidence (r = 0.227, *p* < 0.01) in the enter linear model and is placed second to have the greatest influence on the dementia incidence (increasing R^2^ from 0.728 to 0.738) in the stepwise linear model. Thus, the GDP PPP seems to have less powerful predicting effect on dementia incidence in both enter and stepwise linear models.

**Table 3:** Multiple linear regression analyses to examine predictors of dementia incidence.

Variable	All ages		15–49 years old		
	Beta	Sig	Beta	Sig	
Enter					
*I*_*s*_	-0.620	< 0.001	-0.610	< 0.001	
Ageing	0.257	< 0.001	0.101	0.150	
GDP	-0.010	0.921	0.213	< 0.05	
Urbanization	0.008	0.891	-0.037	0.504	

Stepwise rank	All ages		Rank	15–49 years old	
	Variable	Adjusted R^2^		Variable	Adjusted R^2^
1	*I*_*s*_	0.685	1	*I*_*s*_	0.730
2	Ageing	0.704	2	GDP PPP	0.738

Significance level: *p* < 0.05, *p* < 0.01, *p* < 0.001.

Data sources: Dementia Incidence expressed as the number of new onsets of Alzheimer's disease and other dementias published by the Institute for Health Metrics and Evaluation (IHME) in 2020; *I*_*s*_ measuring the magnitude of the opportunity for natural selection, extracted from the previous publication by You et al. https://doi.org/10.1111/eva.12523; Ageing expressed with life expectancy at 65 years old (2010–2015) published by the United Nations; GDP PPP expressed in purchasing power parity in 2015 US dollars published by the World Bank; Urbanization expressed in percent of population living in urban areas in 2015 published by the World Bank.

Table 4 indicates that, regardless of population grouping criterion, *I*_*s*_ negatively correlates with dementia incidence, although the strength of the correlation and significance levels vary. One of the highlights of the findings is that in developed populations *I*_*s*_ is in the stronger correlation with dementia incidence than it is in less developed population groups. This is supported by the bivariate correlation between *I*_*s*_ and GDP PPP (r = 0.724 and 0.732 in Pearson’s r and Spearman’s rho respectively, Table 1).

**Table 4:** Correlations between I_s_ and dementia incidence in various country groupings.

	All ages		15–49 years old	
	Pearson's r	Non-parametric	Pearson's r	Non-parametric
**WHO region**				
Africa, n = 46	− 0.756***	− 0.556***	− 0.811***	− 0.603***
Americas, n = 35	− 0.759***	− 0.721***	− 0.769***	− 0.736***
Eastern mediterranean, n = 21	− 0.266	− 0.132	− 0.638**	− 0.639**
Europe, n = 50	− 0.639***	− 0.411**	− 0.715**	− 0.691**
South East Asia, n = 11	− 0.502	− 0.373	− 0.682*	− 0.709*
West Pacific, n = 27	− 0.754***	− 0.756***	− 0.763***	− 0.819***
**The World Bank income**				
High, n = 61	− 0.554***	− 0.553***	− 0.414***	− 0.414***
Upper middle, n = 53	− 0.685***	− 0.660***	− 0.726***	− 0.646***
Low middle, n = 48	− 0.788***	− 0.789***	− 0.711***	− 0.752***
Low, n = 28	− 0.712***	− 0.477**	− 0.640***	− 0.386*
**The United Nations Gross National Income (GNI)**				
High, n = 50	− 0.461***	− 0.403**	− 0.351*	− 0.430**
Upper middle, n = 49	− 0.652***	− 0.622***	− 0.695***	− 0.624***
Low middle, n = 44	− 0.760***	− 0.771***	− 0.686***	− 0.732***
Low, n = 31	− 0.710***	− 0.523**	− 0.661***	− 0.467**
**Other country groupings**				
ACD, n = 30	− 0.299	− 0.238	− 0.609***	− 0.638***
APEC, n = 21	− 0.724***	− 0.681***	− 0.532*	− 0.486*
Arab world, n = 22	− 0.142	− 0.026	− 0.630**	− 0.624**
English, official language, n = 49	− 0.839***	− 0.820***	− 0.883***	− 0.872***
LAC, n = 33	− 0.749***	− 0.665***	− 0.768***	− 0.694***
OECD, n = 37	− 0.602***	− 0.327*	− 0.409*	− 0.404*
SADC, n = 16	− 0.770***	− 0.629**	− 0.933***	− 0.779***

Significance level: *p < 0.05; **p < 0.01; *** *p* < 0.001.

Data sources: Dementia Incidence expressed as the number of new onsets of Alzheimer's disease and other dementias published by the Institute for Health Metrics and Evaluation (IHME) in 2020; Opportunity for selection expressed as Is which was extracted from the previous publication by You et al. https://doi.org/10.1111/eva.12523.

*ACD* Asia cooperation dialogue, *APEC* Asia–Pacific economic cooperation, *LAC* Latin America and the Caribbean, *OECD* organisation for economic co-operation and development, *SADC* Southern African Development Community.

## Discussion

Dementia is a growing public health concern owing to multiple aetiologies including population ageing. By assessing the dementia incidence data for 204 populations, this study suggests that less opportunity for natural selection (*I*_*s*_) is another major risk factor for dementia. It is also suggested that people in developed populations have higher risk to develop dementia. In our analyses we used data on the incidence of dementia (new cases per year), however, the results based on these data can also be used, with due caution, to discuss dementia’s prevalence that is another epidemiological variable.

Dementia has strong genetic background which consists of heritable factors directly predisposing to dementia and those causing comorbidities. This has been supported by the studies which concluded that Alzheimer^22^ and frontotemporal^42–45^ dementia are familial diseases. Slooter et al. reported that about ¼ of the people aged 55+ may have genetic predisposition to develop dementia due to their family history of dementia involving their first-degree relatives^46^. Loy et al. conducted a systematic review listing multiple genes involved in the onset of Alzheimer's disease, up to 70% of which may be inherited from a patient's parents^22^. Another review and meta-analysis revealed that the onset of dementia among young people has been on the rise^47^. This may suggest that, due to the reduced natural selection, genetic background of dementia has become more common.

A number of studies have shown that about 20–40% of frontotemporal dementia patients have a positive family history of frontotemporal diseases^42–45^. The strong genetic background of vascular dementia may be attributable to a group of heterogeneous cerebrovascular disorders leading to cognitive impairment^48–50^. For instance, vascular dementia is sometime considered as post-stroke dementia because of the significant association between the nature of vascular dementia and stroke^51,52^, in particular ischemic stroke^53^. Dementia patients usually have a number of comorbidities which are non-communicable diseases. On average, the number of comorbidities of a patient aged 65+ is four, but people without dementia have only two^54^. Most of the comorbidities have genetic predispositions which may be potential triggers for dementia, such as hypertension^55^, depressive disorders^56^, obesity^57^ and diabetes^58^. Therefore, genetic traits of these non-communicable diseases may play a role in increasing predisposition to dementia.

Apparently, numerous genes predispose to dementia, but their unbalanced interactions may also increase dementia incidence in multiple ways because of the pleiotropic effects of those genes. While research into genetic basis for dementia onset is in progress, the identification of specific genes predisposing to specific kinds of dementia remains intriguing and intricate. Dementia genes and their variants are mildly deleterious, and they have not created large potential for damage to human survival^59^. Therefore, they will not be quickly eliminated by natural selection^60,61^. In this study, the inverse correlation of *I*_*s*_ with dementia incidence suggests that relaxed selection has been caused by high level of healthcare services and public health measures in modern society. The actual prevalence of dementias in modern societies will, of course, depend on how well these diseases can be treated by healthcare services.

Globally, high level of healthcare services has relaxed the operation of natural selection in all populations in the past 100–150 years^20^. This was supported by the studies showing that the prevalence rates (total number of people with the condition in the given population) of nasal septa and lacrimal bone defects increased because of the decreasing operation of natural selection^62^. This was also evidenced by the noticeable prevalence of phenylketonuria in a population after this condition had been accumulating for several generations with about 2% increase^20,63^. Recently, the accumulating effects of genetic traits of some diseases have been tested on diabetes^17,21^, obesity^15,16^ and cancers^18^. The inverse correlation of I_s_ with dementia incidence is compatible with alteration of mutation-selection balance.

Without being reduced by modern healthcare services, natural selection could have had an ample opportunity to eliminate the dementia associated defective genetic background introduced by mutations^13,20,64–69^. However, each human life deserves to be protected^20^. Mathematically, heritable dementia incidence could simply be doubled by generation if natural selection were reduced to zero. However, dementia incidence rates are somewhat less than doubled among populations, varying between 421.6 (per 100,000) and 14.0^20,22,23^. Reduced natural selection has led to the greater presence of dementia associated comorbidities with genetic traits, such as diabetes^17,21^, obesity^15,16^ and cancers^18^.

Furthermore, worldwide, in particular in the developed countries, total fertility rate has been decreasing quickly, which has reduced biological variation in fertility^70^. The detrimental effects of decreasing fertility rate have been postulated as the leading risk factor for the increase in breast^41^ and ovarian^40^ cancers. These observations may be applicable to further explore dementia incidence, however, this could form a separate study.

Currently, the clinical treatment for dementias and their comorbidities only focuses on the control of symptoms^71^, but not gene therapy that intends to control the dementia genes/mutation. Clinically, when dementia and its comorbidities are “cured”, it only means that their symptoms, not their genetic background (genes/mutation), are under control or reversed. A recent study has advanced that larger family and household size may be a potential prevention of the dementia mortality^9^. However, to implement this approach the increase in human fertility rate is required which, most certainly, will cause the Earth over-population. Obviously, this is not a practical solution. Currently or for the near future, healthcare services are impotent in removing the genetic traits of dementia and of its comorbidities. For ethical reasons it is impossible to argue that natural selection should be allowed to act on modern human populations. Both, improvements in prevention and cure of dementia symptoms and future developments in gene therapies will hopefully limit detrimental effects of dementias on human life.

## Strengths and limitations

This is an ecological study. Ecological data are based on aggregated quantitative data which can zoom in the rare dementia presence 1,000,000 times, thus dementia presence becomes noticeable for correlating and identifying the potential contributing effects of dementia risk factors at the population level. This necessity has been evidenced in other epidemiological studies of rare non-communicable diseases (cancer^18,40,41^ and Type 1 diabetes^17^).

The intrinsic limitations of ecological studies should be considered when we are examining the public health implications of our results. (1) The results of this study only show the correlation, not causation, of reduced natural selection with dementia incidence. (2) The relationship based on the ecological approach in this work is subject to ecological fallacy. Therefore, the protective role of less reduced natural selection may not always hold true for each individual, but it is possibly true at a population level. (3) The population level data extracted for this study might be fairly crude. The IHME, WHO, United Nations and World Bank may have made some random errors when collecting and aggregating data. (4) The opportunity for natural selection is only measured with respect to postnatal mortality, while gametic selection and intrauterine mortality are not included^72^.

Regardless of the limitations of the ecological data, we have consistently showed that populations with more reduced natural selection had higher dementia incidence. The findings of this study may shed light on further longitudinal studies of human evolution at a population level.

## Conclusion

Worldwide, reduced natural selection may be a significant contributor to the incidence of dementia with special regard to developed populations.

## Data Availability

The data sources have been described in the section of “Material and method”. All data for this study are freely available from the international organizations’ official websites. The formal permission to use the data for non-commercial purpose is not necessary as it is compliant with the agency’s public permission in their terms and conditions.

## Abbreviations

GDP PPP: Gross domestic product (at purchasing power parity)
I_bs_: Biological State Index
*I*_*s*_: A measure of the magnitude of natural selection which was calculated with the formula, *I*_s_ = (1 − *I*_bs_)/*I*_bs_, it is a variance of fitness
Life e(65): Life expectancy at 65 years old
UN: United Nations
WHO: World Health Organization
IHME: Institute for Health Metrics and Evaluation
